# Type V collagen alpha 1 chain promotes the malignancy of glioblastoma through PPRC1-ESM1 axis activation and extracellular matrix remodeling

**DOI:** 10.1038/s41420-021-00661-3

**Published:** 2021-10-26

**Authors:** Hsing-Fang Tsai, Yu-Chan Chang, Chien-Hsiu Li, Ming-Hsien Chan, Chi-Long Chen, Wen-Chiuan Tsai, Michael Hsiao

**Affiliations:** 1 Genomics Research Center, Academia Sinica, Taipei, Taiwan; 2 Department of Biomedical Imaging and Radiological Sciences, National Yang Ming Chiao Tung University, Taipei, Taiwan; 3 Department of Pathology, Taipei Medical University Hospital, Taipei Medical University, Taipei, Taiwan; 4 Department of Pathology, Tri-Service General Hospital, National Defense Medical Center, Taipei, Taiwan; 5 Department of Biochemistry, College of Medicine, Kaohsiung Medical University, Kaohsiung, Taiwan

**Keywords:** CNS cancer, Cell migration, Tumour biomarkers

## Abstract

Glioblastoma (GBM) is a fatal cancer. Existing therapies do not have significant efficacy for GBM patients. Previous studies have shown that the collagen family is involved in the regulation of the extracellular environment of cancer cells, and these conditions could become an important factor for effective treatment. Therefore, we screened various collagen types and observed that the type V collagen α1 chain (COL5A1) gene plays a pivotal role in GBM. We further examined whether the overexpression of COL5A1 is common in mesenchymal subtypes and is related to the survival rate of GBM patients through several in silico cohorts. In addition, our cohort also showed a consistent trend in COL5A1 protein levels. Most importantly, we validated the cell mobility, metastatic ability and actin polymerization status caused by COL5A1 with two-way models. Based on these results, we established a transcriptomics dataset based on COL5A1. Moreover, PPRC1, GK and ESM1 were predicted by ingenuity pathway analysis (IPA) to be transcription factors or to participate downstream. We investigated the involvement of COL5A1 in extracellular remodeling and the regulation of actin filaments in the metastasis of GBM. Our results indicate that the COL5A1−PPRC1−ESM1 axis may represent a novel therapeutic target in GBM.

## Introduction

Glioblastoma (GBM) is a common malignant glioma in the brain and is aggressive [1]. According to the World Health Organization (WHO), Gliomas are divided into low-grade (grades II and III) (Low grade glioma, LGG) and high-grade (grade IV) (GBM) [2]. In addition, brain tumors are the most common metastatic tumors, mainly metastasizing from the lung, breast and urinary system [3, 4]. The other type is primary tumor lesions derived from glial cells, which account for half of all brain tumors and are almost all malignant tumors [5]. At present, the treatment for brain cancer mainly includes surgery, chemotherapy, radiation therapy, immunotherapy, etc. [6, 7]. Temozolomide (TMZ) is the only chemotherapeutic drug that has been approved by the US Food and Drug Administration (FDA) for the treatment of GBM. In recent years, scientists have tried to classify the subtypes and genetic alterations [8]. They defined the proneural, classical and mesenchymal subtypes and multiple corresponding events. These events include the IDH1 R132 mutation, CpG island methylator phenotype (CIMP) status, methylation state of MGMT, ATRX loss-of-function mutations, EGFR amplification, and several chromosomal abnormalities (chromosome 1p-19q codeletion, chromosome 7 gain-of-function and chromosome 10 loss-of-function) [9, 10]. Although the incident and pathological background have been described, the detailed mechanisms of the extracellular environment are still unknown.

Collagen is a polypeptide that accounts for approximately one-third of the extracellular matrix (ECM) protein of connective tissue in the human body [11, 12]. Based on structural and functional properties, collagen is divided into 28 types that have been identified [13, 14]. Type V collagen is classified as fibrillary collagen along with types I−XI. In many connective tissues, type V collagen is coexpressed with type I collagen, but there is a low level of type V collagen [15]. Collagen V obviously comes from one or more thin fibers of the same type, and many collagens are involved in the establishment of the physical properties of such fibers [16]. Collagen fibers and proteoglycans constitute the structure and are involved in the remodeling of the ECM [17]. Proteoglycans rely on core proteins and glycosaminoglycans (GAGs) to support their biological function, including chondroitin sulfate (CS), keratin sulfate (KS), dermatan sulfate (DS) and heparin sulfate (HS) [18, 19]. Endothelial cell-specific molecule-1 (*ESM1*, Endocan) has been described and is secreted in the form of soluble DS proteoglycans [20]. ESM1 can modulate the activity of growth factors, chemokines, and coagulation factors, thereby further causing inflammation, hemostasis and angiogenesis [21].

Recent studies have found that COL5A1 may be related to the occurrence and progression of several types of malignant tumors. Several comprehensive bioinformatics analyses also showed that COL5A1 is one of the key genes that differentiates inflammatory and noninflammatory breast cancer [22]. However, the detailed molecular mechanism and phenotypic regulation of COL5A1 in GBM have not yet been investigated. In our study, we systematically screened the association between the expression levels of collagen family members in online available datasets (TCGA and array-based chips). Our results showed that COL5A1 expression in GBM is higher than that in normal adjacent tissues. The results of immunohistochemical (IHC) staining were similar. The protein expression of COL5A1 is highly correlated with staining, poor survival rates and various genetic alterations in GBM. In regard to cell function, we proved that COL5A1 can promote cell migration/invasion and actin polymerization. In contrast, we also observed that the downregulation of COL5A1 expression in tumor cells led to a functional phenotype reversal. Furthermore, we investigated the potential transcriptional regulator PPRC1 and its downstream target ESM1 in our established COL5A1 transcriptomic profiles. Finally, we confirmed that the neutralizing antibody and recombinant protein of ESM1 can reverse the phenotype induced by COL5A1. Based on all of the evidence, our data suggest that COL5A1 may be a novel prognostic target and that the COL5A1−PPRC1−ESM1 axis is a promising therapeutic target for malignant glioblastoma.

## Results

### COL5A1 expression is associated with various genetic events and survival rates in GBM

**Fig. 1:**
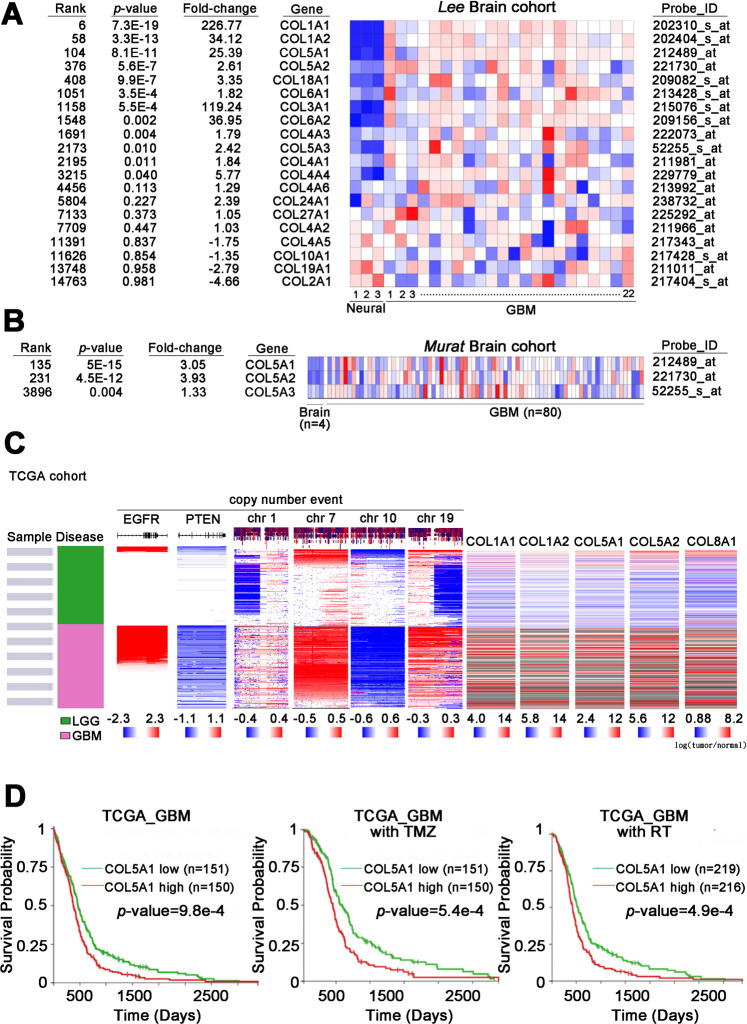
COL5A1 mRNA expression in various subtypes of glioma and its correlations with several events. **A** The heatmap shows the correlation between the mRNA expression levels of various collagen family members in neural/GBM tissues from Oncomine (Lee brain cohort, *n* = 25). **B** The heatmap shows the correlation between the mRNA expression level of collagen V family members in normal brain/GBM tissues from Oncomine (Murata brain cohort, *n* = 84). **C** The heatmap shows the endogenous mRNA expression levels of several targets, genetic alteration events, and subtypes in the TCGA-specific template (copy number for EGFR, PTEN, and chromosomes 1, 7, 10, and 19 in TCGA brain tumors, *n* = 1119). **D** Kaplan–Meier curves of overall survival in glioblastoma patients with or without TMZ treatment and radiotherapy based on a high or low COL5A1 mRNA expression level (*p* = 9.8e−4, *p* = 5.4e−4 and *p* = 4.9e−4, respectively). The significance of the differences in (**D**) was analyzed using Student’s *t* test.

To examine the role of the collagen family in brain tumors and identify which family members have a unique effect, we utilized in silico data available on the Oncomine website. In the *Lee* brain cohort (*n* = 25), we observed that several collagen family members were increased in GBM compared to normal neural tissues, including type I (COL1A1 and COL1A2) and type V (COL5A1-COL5A2) (Fig. 1A). Previously, the biological functions and mechanisms of the type I collagen family have been identified, but type V collagen has been studied little. We further analyzed the type V collagen family (COL5A1−COL5A3) in a validation cohort (the *Murate* brain cohort, *n* = 80). Our results showed that COL5A1 had the highest ranking, with significantly higher expression levels in GBM tissues than normal brain tissues (Fig. 1B). Not only were the RNA expression levels of the collagen family compared in these collected populations, but our candidate genes were also examined in TCGA. We generated a comprehensive panel based on clinical parameters, genetic alterations and target gene expression levels in brain tumors (low-grade glioma and glioblastoma). Several events (the chromosome 1/7/10/19 status, PTEN loss-of-function mutations and EGFR amplification) were included as the classification criteria to differentiate LGG and GBM. Our results clearly showed that COL5A1 changed significantly in the GBM group (Fig. 1C). After quantifying the expression level of COL5A1 in samples with several events, we confirmed that COL5A1 and the existing parameters that define GBM have clear *p* values (Supplementary Fig. 1). We further determined that COL5A1 was overexpressed in samples with several parameters, including the non-G-CIMP, IDH1 wild-type and unmethylated MGMT groups (*p* < 0.0001, *p* < 0.0001 and *p* = 0.008, respectively) (Supplementary Fig. 2). In regard to the molecular subtype classification of GBM, the expression level of COL5A1 in the mesenchymal type was higher than that in the proneural and classical subtypes (*p* < 0.001). There was no significant difference between male and female patients (*p* = 0.1215).

Next, we examined the effect of the COL5A1 expression level on the survival rate of TCGA GBM patients. We found that COL5A1 was positively correlated with the overall survival of patients (*p* = 9.8e−4) (Fig. 1D). Interestingly, we examined the COL5A1 performance and overall survival rate of patients treated with temozolomide (TMZ) and radiation therapy (RT). These Kaplan–Meier plots also showed that COL5A1 was a prognostic factor for survival in GBM patients (*p* = 5.4e−4 and *p* = 4.9e−4, respectively) (Fig. 1D). Based on these findings, we observed that COL5A1 is related to many clinical indicators, and its uniqueness is suitable for the in-depth analysis in GBM.

### COL5A1 has prognostic value and is a high risk factor for glioma

**Fig. 2:**
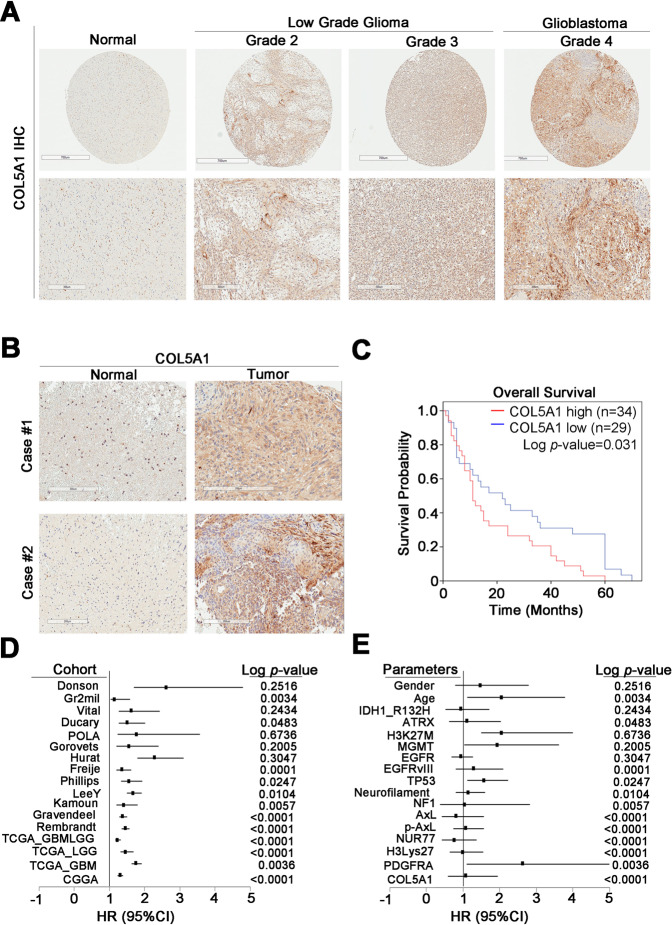
Overexpression of COL5A1 correlates with poor survival in glioma patients. **A** Scores (0–3) indicate COL5A1 levels in representative glioblastoma tumor tissues. **B** Expression level of the COL5A1 protein in tumor tissue compared to that in corresponding normal adjacent tissue. **C** Kaplan–Meier overall survival curve of 63 glioma patients stratified according to a high or low COL5A1 protein expression level (*p* = 0.031). **D** A forest plot was generated with the hazard ratio and 95% confidence interval (CI) of the COL5A1 expression level in various clinical cohorts. **E** A forest plot was generated with the hazard ratio and 95% confidence interval (CI) of the COL5A1 expression level according to various clinicopathological factors in an existing cohort. The significance of the differences in (**C**) was analyzed using Student’s *t* test.

The protein levels of COL5A1 showed a consistent trend. We performed immunohistochemical (IHC) staining with an anti-COL5A1 antibody in our glioma cohort. After IHC scoring, we observed that COL5A1 was increased at the protein level in low-grade glioma and GBM patients (Fig. 2A). In several normal/tumor pairs (*n* = 5), the expression of COL5A1 in the tumor tissues was higher than that in the normal adjacent tissues (Fig. 2B). We found that COL5A1 expression was correlated with poor survival in glioma patients (*p* = 0.031) (Fig. 2C). We further measured the hazard ratio (HR) and Cox *p* value of each clinicopathological factor in the glioma tissue microarray (TMA) with univariate and multivariate analysis (Fig. 2D and Tables 1 and 2). We evaluated the association of COL5A1 expression with some available prognostic factors, including IDH1 mutation, ATRX loss-of-function mutations and neurofilaments. Surprisingly, we found that COL5A1 can not only be used as an independent prognostic factor (HR = 1.487, *p* < 0.0001) but can also compete with several existing factors. These markers included H3K27M, MGMT methylation states and EGFR amplification (*p* = 0.6736, *p* = 0.2005, and *p* = 0.3047, respectively) (Fig. 2E). These data indicate that the overexpression of COL5A1 has prognostic value, and the mechanism should be further studied.

**Table 1:** Univariate analysis of COL5A1 expression and clinical parameters in gliomas.

	Univariate analysis		
Variable	Hazard ratio	95% confidence interval	*p* value
Sex (male/female)	1.077	0.594–1.953	0.806
Age (<65/≥65)	2.626	1.110–6.216	0.028*
IDH1 R132H (negative/positive)	1.000	0.647–1.547	0.999
ATRX (preserved/lost)	0.771	0.428–1.387	0.385
H3 K27M (negative/positive)	1.084	0.747–1.572	0.671
MGMT (preserved/lost)	0.816	0.426–1.565	0.541
EGFR (negative/positive)	1.038	0.382–2.819	0.942
EGFRvIII (negative/positive)	1.138	0.807–1.604	0.462
P53 (negative/overexpressed)	1.576	1.118–2.220	0.009*
Neurofilament (negative/positive)	1.296	0.805–2.087	0.286
NF1 (negative/positive)	0.938	0.697–1.263	0.673
PDGFRA (negative/positive)	2.054	1.117–3.777	0.021*
COL5A1(negative/positive)	1.487	0.792–2.792	0.218

*A *p* value < 0.05 was considered significant.

**Table 2:** Multivariate analysis of COL5A1 expression and clinical parameters in gliomas.

	Multivariate analysis		
Variable	Hazard ratio	95% confidence interval	*p* value
Sex (male/female)	2.147	0.958–4.812	0.064
Age (<65/≥65)	8.168	1.923–34.698	0.004*
IDH1 R132H (negative/positive)	1.202	0.699–2.066	0.506
ATRX (preserved/lost)	1.203	0.507–2.853	0.675
H3 K27M (negative/positive)	0.979	0.597–1.606	0.933
MGMT (preserved/lost)	0.555	0.252–1.221	0.143
EGFR (negative/positive)	0.637	0.195–2.081	0.455
EGFRvIII (negative/positive)	0.877	0.531–1.447	0.607
P53 (negative/overexpressed)	1.438	1.065–2.142	0.024*
Neurofilament (negative/positive)	2.054	1.015–4.153	0.045*
NF1 (negative/positive)	0.693	0.419–1.147	0.154
PDGFRA (negative/positive)	2.285	1.005–5.194	0.049*
COL5A1(negative/positive)	1.260	1.522–3.038	0.047*

*A *p* value < 0.05 was considered significant.

### COL5A1 promotes migration, invasion and actin polymerization

**Fig. 3:**
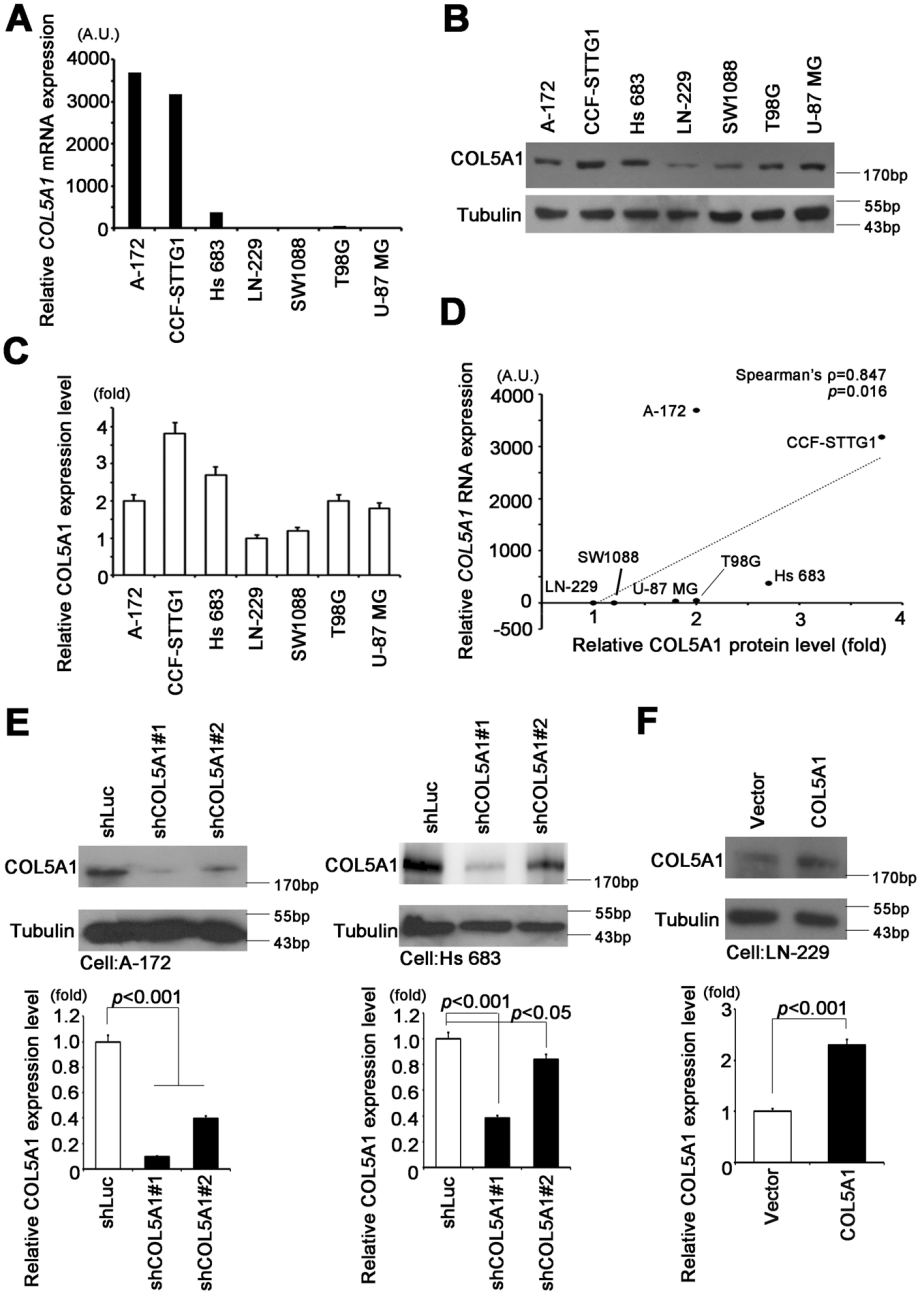
COL5A1 expression is positively correlated with metastasis ability in GBM cells. **A** qRT-PCR analysis of COL5A1 expression in various GBM cell lines. **B** Western blot analysis of COL5A1 and tubulin protein expression in the GBM cell panel. **C** Quantitative results of COL5A1 in the GBM cell panel. **D** Correlation plot indicating the relationship between COL5A1 mRNA and protein expression levels (Spearman’s rho = 0.733, *p* = 0.025). **E** Western blot analysis and quantitative results of COL5A1 knockdown in A-172 and Hs 683 cells. **F** Western blot analysis and quantitative results of COL5A1 overexpression in LN-229 cells. Tubulin was used as an internal control for protein loading. The significance of the difference was analyzed using the nonparametric Mann–Whitney *U* test. The AU represents absorbance units.

**Fig. 4:**
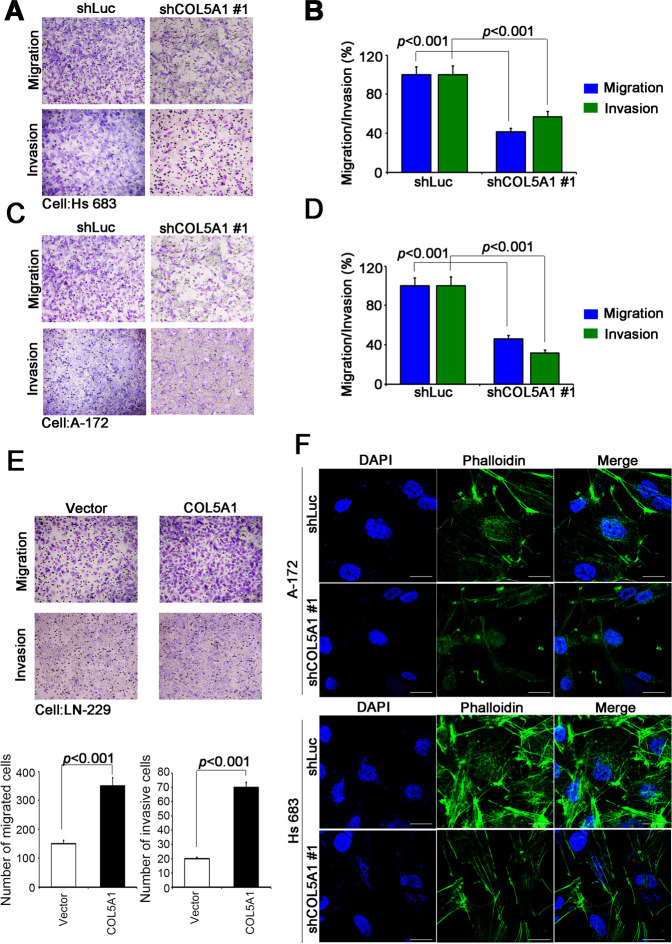
COL5A1 regulates the migration/invasion ability and actin polymerization of GBM cells. **A** The migration and invasion abilities of Hs 683 cells after COL5A1 knockdown clone #1. **B** Quantitative results of Hs 683 cell migration and invasion ability after COL5A1 knockdown clone #1. **C** The migration and invasion abilities of A-172 cells after COL5A1 knockdown clone #1. **D** Quantitative results of A-172 cell migration and invasion ability after COL5A1 knockdown clone #1. **E** The migration and invasion abilities and quantitative results of LN-229 cells overexpressing the exogenous COL5A1 gene. **F** Immunofluorescence assay of A-172 and Hs 683 cells after COL5A1 knockdown clone #1. Green, phalloidin; blue, DAPI. The significance of the difference was analyzed using the nonparametric Mann–Whitney *U* test.

To determine the appropriate cell model for phenotype validation, we screened the endogenous COL5A1 mRNA level in a GBM cell panel (Supplementary Fig. 3). We collected A-172, CCF-STTG1, Hs 683, LN-229, SW1088, T98G and U-87 MG cells for qRT-PCR and western blot analysis. We observed that A-172, CCF-STTG1 and Hs 683 cells had high COL5A1 expression levels at both the mRNA and protein levels (Fig. 3A−C). Our results also showed that the RNA and protein levels of COL5A1 were closely related in GBM cell lines (Spearman’s rho = 0.733, *p* value = 0.025) (Fig. 3D). Therefore, we measured the knockdown efficiency of two independent clones of COL5A1 shRNA in A-172 and Hs683 cells. On the other hand, we established a stable COL5A1 overexpression model in LN-229 cells (Fig. 3F). We used these stable cells for functional analysis and found that COL5A1 regulates cell migration and metastasis. With Boyden chamber assays, we confirmed that Hs 683 cells have high migration and invasion capabilities. However, when COL5A1 was downregulated in the Hs 683 cell line, cell migration/invasion was significantly reduced (Fig. 4A, B). In addition, we used a secondary COL5A1 knockdown model in A-172 cells. The results were also similar to those of the Hs 683 group (Fig. 4C, D). To confirm that the phenotype was not due to shRNA off-target effects, we also analyzed LN-229 COL5A1-overexpressing cells in a complementary model. Indeed, the COL5A1 overexpression group had increased migration and invasion abilities in LN-229 cells (Fig. 4E). Previous studies reported that cell mobility and migration may be the result of cytoskeletal remodeling and actin filament rearrangement [23]. Therefore, we used the actin-specific florescent dye phalloidin to stain our COL5A1 knockdown models. Combining phalloidin and DAPI imaging, we found that COL5A1 participated in the maintenance of actin filaments and the cytoskeleton (Fig. 4F). Thus, we proved that COL5A1 is related to GBM cell migration ability and actin polymerization.

### ESM1 regulates actin polymerization and cell metastasis through the COL5A1−PPRC1 axis

**Fig. 5:**
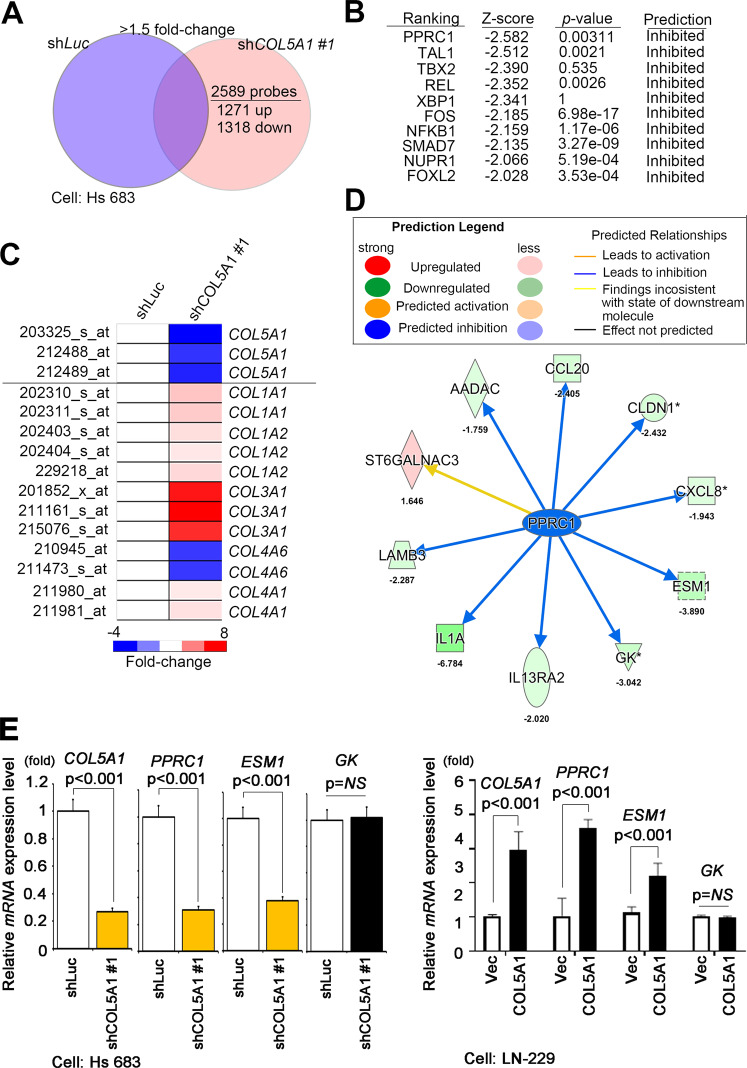
COL5A1 modulates the activity of PPRC1 and its downstream target ESM1. **A** Venn diagram illustrating that when comparing Hs 683 shCOL5A1 #1 and Hs 683 shLuc cells, 2589 probes with a cutoff value >1.5-fold change were identified. **B** Ranking of the upstream transcriptional regulators of predicted candidate genes according to the IPA database of the Hs 683 shCOL5A1 microarray compared to the Hs 683 shLuc microarray. **C** The heatmap shows the expression levels of collagen family members in the available chips. **D** The network was predicted using the selection signature that was overlaid with microarray data from Hs 683 shCOL5A1 cells with a 1.5-fold change cutoff in the IPA database. The intensity of the node color indicates the degree of activating (orange) or inhibiting (blue) regulation following COL5A1 interactions. **E** The downstream targets of PPRC1 were analyzed by qRT-PCR in the Hs 683 COL5A1-knockdown model and the LN-229 COL5A1-overexpression model. The significance of the difference was analyzed using the nonparametric Mann–Whitney *U* test.

To reveal the potential molecular mechanism of COL5A1 in GBM metastasis, we established COL5A1-based transcriptome profiles through microarray chips. A total of 2589 probes were identified, and the knockdown group had a >1.5-fold change compared to the vector control. These probes included 1271 upregulated and 1318 downregulated probes selected with GeneSpring software (Fig. 5A). These candidates were predicted to be potential canonical pathways and upstream regulators with the Ingenuity Pathway Analysis (IPA) tool. Our interpretation list included ten significant *z*-score signatures and PPRC1, which implied that they were inhibited in the COL5A1 knockdown model (Fig. 5B). To determine whether COL5A1 acts in coordination or is redundant with other collagen family members in the GBM study, we examined the type I−V collagen members in our datasets. Except for COL4A6, most collagen members were overexpressed in COL5A1-knockdown cells (Fig. 5C). Therefore, we hypothesized that COL5A1 may regulate GBM cell migration and invasion ability through its unique role. In addition, we verified the downstream factors of PPRC1 in the COL5A1 two-way models (Fig. 5D). Our qRT-PCR data prove that *ESM1* expression is more dominant than the expression of other genes (Fig. 5E and Supplementary Fig. 4).

**Fig. 6:**
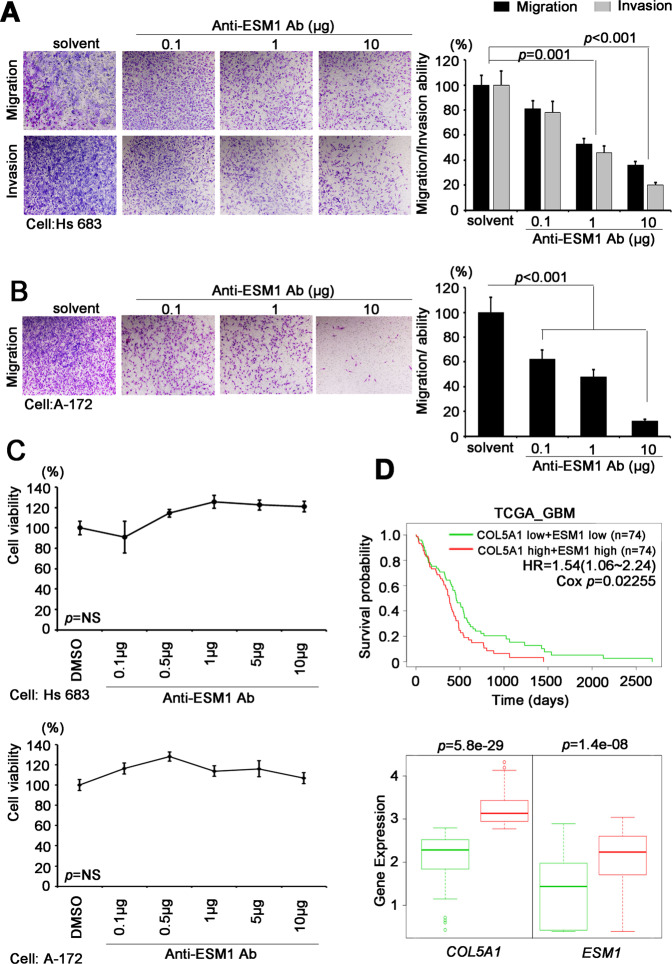
ESM1 acts as the coordinator of the COL5A1-induced phenotype in GBM. **A** Migration and invasion abilities and quantitative results of the ESM1-neutralizing antibody in Hs 683 cells. **B** Migration ability and quantitative results of the ESM1-neutralizing antibody in A-172 cells. **C** The viability of Hs 683 and A-172 cells with or without ESM1-neutralizing antibody treatment. **D** Kaplan–Meier plot.

**Fig. 7:**
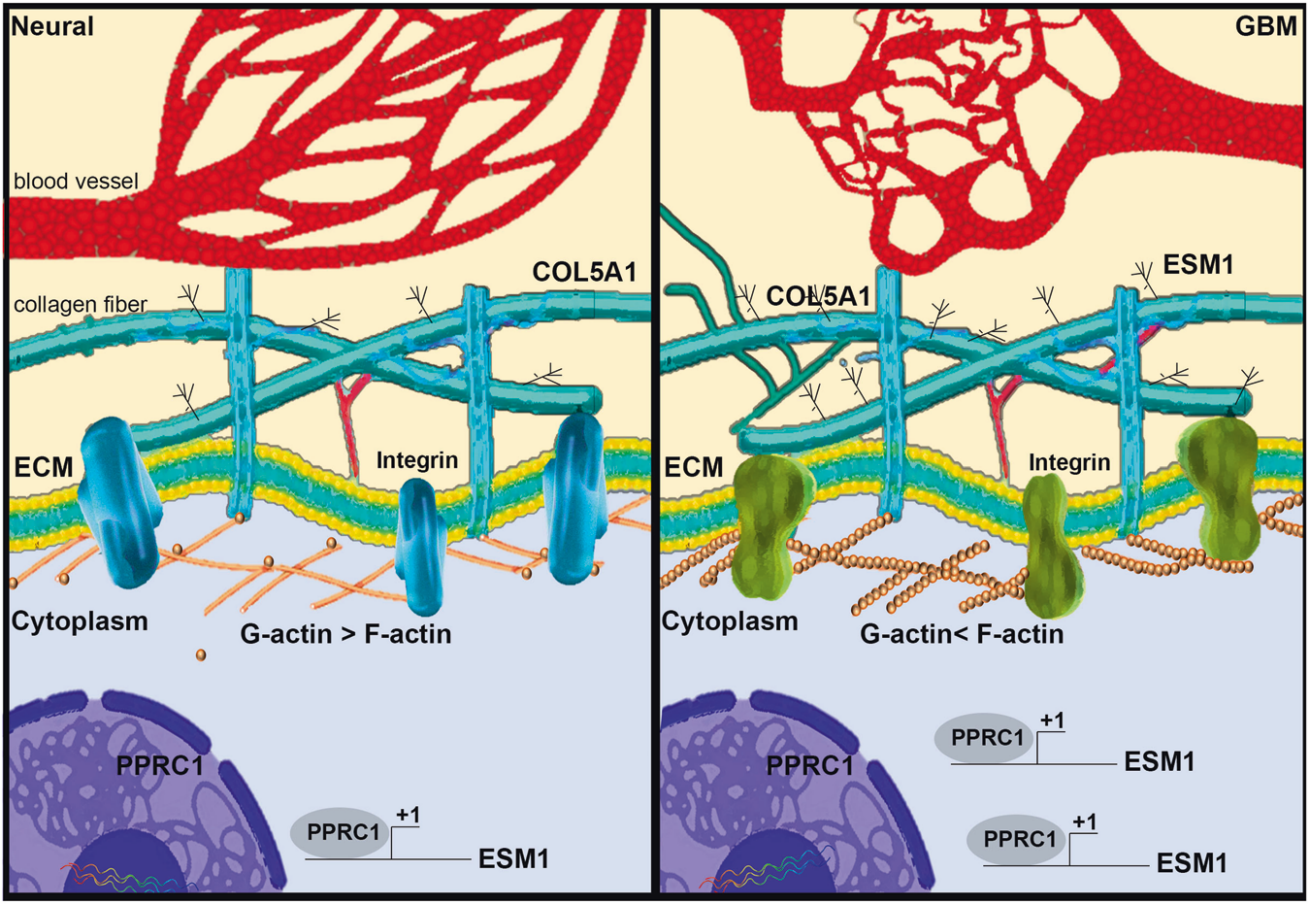
Schematic model of the COL5A1−PPRC1−ESM1 axis in GBM tumorigenesis. COL5A1 can reprogram the cytoskeleton composition, thereby upregulating the ratio of F-actin, and promoting PPRC1-ESM1 axis to accelerate cell migration, and invasion.

To confirm the importance of ESM1 in our study, we then used neutralizing antibodies against ESM1 in highly aggressive GBM cells. We observed that neutralizing antibodies reduced migration/invasion ability in a dose-dependent manner (Fig. 6A, B). More importantly, ESM1-neutralizing antibodies inhibited migration/invasion ability, while proliferation and viability were not affected (Fig. 6C). In a clinical setting, we also speculated about the relationship between COL5A1 and ESM1. Therefore, we examined the agreement between ESM1 and various PPRC1 downstream genes (*CLDN1,* *CCL20,* *ESM1,* *LAMB3, ST6GALNAC6* and *GK*) (Supplementary Fig. 5). We found that *ESM1* had the highest correlation with COL5A1 in GBM patients (*R* = 0.54, *p* = 1.5–13) (Fig. 6D and Supplementary Fig. 5). Finally, we identified an important axis by which COL5A1 triggers PPRC1 activity and turns on the downstream target ESM1. Then, GBM cells acquire the ability to metastasize, and the cytoskeleton/ESM is remodeled (Fig. 7).

## Discussion

COL5A1 is a gene that encodes a low-abundance type V fibrous collagen alpha chain [24]. Fibrous collagen molecules are trimers that can be composed of one or more types of alpha chains [25]. Similar to COL5A1, it has been previously reported that type V collagen can assemble heterotypic fibers with type I collagen members [26]. However, the detailed pathogenesis and mechanism of COL5A1 are still unclear [27, 28]. In recent years, various molecular biomarkers and genetic events have been used for classification and prognosis, which has continuously improved the accuracy of brain cancer treatment [29]. In our study, we examined several available cohorts and found that the expression level of COL5A1 is related to certain clinicopathological parameters and even to the survival rate of GBM patients. We screened a large number of collagen fiber family members and observed that COL5A1 plays a unique role in GBM. Through our COL5A1-based transcriptomics profiles, we found that when COL5A1 was knocked down, the expression level of most members of the fibrous collagen family was not affected. For these underlying mechanisms and additional phenotypes of COL5A1, we will further investigate and reveal the actual situation. All this evidence indicates that COL5A1 plays a dominant role in the composition of fibrous collagen, extracellular matrix remodeling and the cancer microenvironment. Moreover, COL5A1 may reflect the clinical features and prognostic value of GBM.

Last year, The Consortium to Inform Molecular and Practical Approaches to CNS Tumor Taxonomy—Not Official WHO (cIMPACT-NOW) was formed in late 2016 by a group of neuropathology and neuro-oncology experts to provide practical recommendations (published as cIMPACT-NOW updates) [30]. Several subtypes and events have been reevaluated and reclassified, including the following. (1) The definitions of “not otherwise specified, (NOS)” and “not elsewhere classified (NEC)” were reevaluated. (2) Some molecular biomarkers were adjusted or excluded. (3) Subtype classifications were changed. (4). Novel genetic alterations for prognostic/diagnostic applications were identified [31–38]. Changes in these rules will affect the state and distribution of the samples we collect. Currently, we are reassessing whether COL5A1 expression also meets the latest criteria as a prognostic/diagnostic factor for glioma and glioblastoma if the updated version of WHO classification of CNS tumors is formally published.

Members of the collagen family have been reported to be dysfunctional in various cancers or diseases. However, whether there are interactions between family members remains unknown. Previous studies have observed their common characteristics, so it is necessary to explore their coordination or redundancy. In our microarray datasets, we also observed the upregulation of different family members when COL5A1 was inhibited. We will use a CRISPR approach to understand whether the specific targets are compromised or reprogrammed in the collagen system. On the other hand, we will determine whether there is a significant difference in the IC50 of TMZ in our cell model. If we examine the differences, the research will continue to explore the possible signaling pathways and whether autophagy, stemness, and apoptosis are affected by COL5A1. In line with our transcriptomics data, we can examine multiple possible pathways as a direction for drug testing. In addition, the synergistic effects will be evaluated with available drug options (e.g., TMZ). These observations may be applied in animal models and preclinical trials. We anticipate that the survival rate of GBM patients will be increased.

## Conclusions

In this study, we examined the function of COL5A1 in the TCGA database and various in silico cohorts. We confirmed that COL5A1 is related to certain genetic events, clinicopathological parameters and survival patterns. We also determined the protein level of COL5A1 and examined its use as a prognostic factor for tumor staging in brain tumor patients. We further established two types of stable COL5A1 cell lines by knocking down and overexpressing it to examine the invasion, migration and cytoskeletal remodeling of GBM cells. Moreover, we demonstrated that COL5A1 can activate PPRC1 transcriptional activity and its downstream target ESM1. By regulating ESM1 expression, the COL5A1-induced phenotype was reversed. Furthermore, we discovered the translational clinical value and clinicopathological effects of the COL5A1−PPRC1−ESM1 axis. Affecting the interplay between collagen and extracellular remodeling will be a novel therapeutic method for GBM.

## Materials and methods

### In silico study

The clinical data and genome matrix from the Cancer Genome Atlas (TCGA) were downloaded from the USCS Xena browser website (https://xenabrowser.net/heatmap/). All GSE datasets were downloaded from the Gene Express Omnibus (GEO) website, normalized and analyzed with GeneSpring software (Version 13.1.1, Agilent, Santa Clara, CA, USA). All data that we downloaded included the expression level and clinical parameters of target genes in glioma patients from the Xena browser. For genes with higher expression, the median was set to be higher than that for genes with high expression and vice versa. Additionally, we excluded several clinical cases that lacked the corresponding parameters. Statistical analyses were performed using SPSS 17.0 software (SPSS, Inc., Chicago, IL, USA). Differences between the two groups were analyzed using a paired *t* test or a Mann–Whitney *U* test. *p* values lower than 0.05 were considered statistically significant.

### Case selection

From 1997 to 2005, a total of 76 patients were diagnosed with different types of glioma at the Tri-Service General Hospital in Taiwan. All the included patients were reclassified as follows: 3 diffuse astrocytoma with mutant IDH, 1 pilocytic astrocytoma, 7 diffuse astrocytoma with wild-type IDH, 6 anaplastic astrocytoma with wild-type IDH, 3 anaplastic astrocytoma mutant with IDH, 3 glioblastomas with mutant IDH, 24 glioblastomas with wild-type IDH, 11 diffuse midline gliomas, 2 oligodendrogliomas (H3 K27M-mutant, NOS) and 4 anaplastic oligodendrogliomas NOS based on immunohistochemical staining. No patient had ever received radiation therapy or preoperative chemotherapy. The clinical information and pathology data were collected via a retrospective review of patient medical records. All patients were diagnosed according to the fourth version of the World Health Organization (WHO) classification of Tumours of the Central Nervous System (2016). Tracking data were available for all patients, and the longest clinical follow-up period was 60 months. Written informed consent for the biological studies was obtained from each patient involved in the study, and the study was approved by the Institutional Review Board (IRB) of Tri-Service General Hospital (098-05-295).

### TMA immunohistochemistry stain assay and interpretation

Representative 1-mm-diameter cores from each tumor taken from formalin-fixed paraffin-embedded tissue were selected based on having morphology typical of the diagnosis. The histopathologic diagnosis of all samples was reviewed and confirmed by a pathologist with hematoxylin and eosin-stained slides. IHC staining was performed on serial 5-µm-thick tissue sections cut from a TMA using an automated immune stainer (Ventana Discovery XT autostainer, Ventana Medical Systems, Tucson, AZ, USA). Sections were first dewaxed in a 60 °C oven, deparaffinized in xylene, and rehydrated in graded alcohol. Antigens were retrieved by heat-induced antigen retrieval for 30 min with pH 8.0 TRIS-EDTA buffer. Slides were stained with a polyclonal rabbit anti-human COL5A1 antibody (HPA030769, 1:1000, Atlas).

The intensity of IHC staining was scored by a pathologist as follows: a score of 0 was defined as no cytoplasmic staining or cytoplasmic in <10% of tumor cells; a score of 1+ was defined as weak/almost imperceptible partial cytoplasmic staining in >10% of tumor cells; a score of 2+ was defined as moderate cytoplasmic staining in >10% of tumor cells; and a score of 3+ was defined as strong cytoplasmic staining in >10% of tumor cells. Low COL5A1 expression was defined as scores of 0 and 1+; otherwise, scores of 2+ and 3+ were defined as high COL5A1 expression.

### Cell line and cell culture

The human brain cancer cell lines A-172, Hs 683, and LN-229 were cultured in Dulbecco’s Modified Eagle Medium (DMEM). SW1088 was cultured in L-15. T98G and U-87 MG were cultured in Minimum Essential Media (MEM). CCF-STGG1 was cultured in Roswell Park Memorial Institute (RPMI). All the culture media were supplemented with penicillin−streptomycin−glutamine (GIBCO; final concentrations: 100 units of penicillin, 100 µg streptomycin, and 0.292 mg glutamine per milliliter of medium) and 10% Fetal Bovine Serum (FBS) (Invitrogen). All cells were incubated in a 37 °C incubator in an atmosphere containing 5% CO_2_. All cell lines were purchased from the American Type Culture Collection (ATCC).

### Lentiviral infections and shRNA sequences

Lentiviral COL5A1 shRNA constructs were purchased from the National RNAi Core Facility Laboratory (Academia Sinica, Taiwan). Lentiviruses were produced by cotransfecting the shRNA expression vector with the pMDG and pΔ8.91 constructs into 293T cells using calcium phosphate. Viral supernatants were then harvested and used to infect A-172 and Hs 683 cells in the presence of 8 μg/mL polybrene (Santa Cruz). Cells were then selected using 2 μg/mL puromycin (Santa Cruz). COL5A1-expressing cells were established by infecting cells with the pLenti6.3-COL5A1 virus, and viral supernatants were harvested and used to infect LN-229 cells in the presence of 8 μg/mL polybrene. Cells were selected using 5 μg/mL blasticidin (Sigma).

### Boyden’s chamber migration/invasion assay

The migration experiment used polycarbonate filters (GE Healthcare Life Sciences, Chalfont St. Giles, UK) and used a concentration of 1 mg/mL human fibronectin (Sigma, St. Louis, MO, USA). Cell culture medium containing 10% FBS was added to each well in the lower area of the Boyden chamber. A total of 1.5 × 10^4^ cells were resuspended in a serum-free culture medium and seeded into each well corresponding in the upper part of the Boyden chamber. In the invasion experiment, a concentration of 1 mg/mL human fibronectin was used, and the other side was attached to 10 mg/mL Matrigel (BD Biosciences, San Jose, CA, USA). Cell culture medium containing 10% FBS was added in the well in the lower area of the Boyden chamber. The cells were resuspended in a serum-free culture medium and loaded into each well in the upper area of the Boyden chamber. After a set length of time (migration: 8 h, invasion: 14 h), the insert was collected, and the cells were stained with Giemsa solution and counted under an optical microscope (×400, 8 random fields per well). The experiment was performed with three independent experimental replicates and four replicates for each sample.

### Real-time PCR gene amplification analysis

Five micrograms of RNA was extracted from cancer cells, and then reverse transcription was performed with Superscript III reverse transcriptase (Invitrogen-Gibco, NY). COL5A1 expression was examined by a UPL system (Roche, Switzerland) with specific primers and normalized to GAPDH expression. Other gene expression assays were performed with OmicsGreen (Omics Bio, Taipei, Taiwan).

### Western blot assay

Total protein lysate was harvested from variant culture cell lines with lysis buffer containing proteinase inhibitors on ice to detect endogenous proteins. After centrifugation, 40 μg protein lysate in loading buffer was loaded in each lane. Proteins were separated and transferred with standard procedures for western blotting. After blocking and incubation with primary antibodies against COL5A1 (HPA030769, 1:1000, Atlas) and β-actin (A2228, 1:5000, Sigma), the luminescence signals were detected with ECL Pro reagent (PerkinElmer) after HRP-antibody hybridization. The images were recorded with an LAS-3000 Imaging System (Fuji).

### Microarray analysis

Total RNA was extracted from the cell line using an RNeasy Mini kit (Qiagen, Valencia, CA, USA) and analyzed with a 2100 Bioanalyzer (Agilent Technologies, Palo Alto, CA, USA) to evaluate whether the RNA quality met the requirements of the microarray chip. RNA was quantified and then applied to a GeneChip 3ʹ IVT Expression Kit & Hybridization Wash and Stain Kit (Affymetrix, Santa Clara, CA, USA). The RNA was loaded onto Affymetrix GeneChip Human Genome U133 plus 2.0 arrays (Affymetrix, Santa Clara, CA, USA) chip. All gene-related probes were analyzed, and Log2-standardized with GeneSpring (Agilent Technologies, Palo Alto, CA, USA). We further analyzed gene probes with >1.5-fold increases/decreases in COL5A1-knockdown cells (clone #1) compared with the Hs 683 cells in the control group (*n* = 1). We confirmed that shCOL5A1 #1 versus control and shCOL5A1 #2 versus control had consistent changes. The probes selected from the array also confirmed this change using qPCR and multiple stable cell models. Furthermore, we predicted the potential upstream transcription factors and signaling pathways with IPA (Qiagen, CA, USA) online tools. These microarray data were uploaded to the National Center for Biotechnology Information (NCBI) Gene Expression Omnibus (GEO).

### Phalloidin staining

The cells (2 × 10^4^) were cultured in a 12-well plate on a glass slide. The cells were fixed with 3.7% formaldehyde for 15 min at room temperature. The cells were rinsed twice with Phosphate Buffered Saline (PBS), and 0.1% X-Triton 100 (Sigma, St. Louis, MO, USA) was used to permeabilize the sample for 15 min. The sample was rinsed twice with PBS. Phalloidin (Invitrogen, Carlsbad, CA, USA) was diluted 40 times with methanol, and 1% Bovine Serum Albumin (BSA) was added to prevent background generation. Diluted phalloidin was added to the sample and allowed to react at room temperature for 1 h. After washing twice with PBS, the membrane was covered with a solution containing 4’,6-diamidino-2-phenylindole (DAPI) (Thermo, Waltham, MA, USA).

### Statistical analysis

The nonparametric Mann–Whitney *U* test was used to analyze the statistical significance of the results from three independent experiments. Statistical analyses were performed using SPSS (Statistical Package for the Social Sciences) 17.0 software (SPSS, Chicago, IL, USA). The association between clinicopathological categorical variables and COlL5A1 IHC expression levels was analyzed by Pearson’s chi-square test. Estimates of the survival rates were calculated using the Kaplan–Meier method and compared using the log-rank test. Follow-up time was censored if the patient was lost during follow-up. Univariate and multivariate analyses were performed using Cox proportional hazards regression analysis with and without adjustments for COL5A1 IHC expression level and grade. For all analyses, a *p* value of <0.05 was considered significant.

## Supplementary information

Supplementary information

## Data Availability

The data that support the findings of this study are available from the corresponding authors upon reasonable request.
