# Supplementary material for: Type V collagen alpha 1 chain promotes the malignancy of glioblastoma through PPRC1-ESM1 axis activation and extracellular matrix remodeling
Source: Cell Death Discov. 2021 Oct 26;7:313. doi: 10.1038/s41420-021-00661-3 (PMC8548600; doi:10.1038/s41420-021-00661-3)

**Supplementary information**

**Type V Collagen Alpha 1 Chain Promotes the Malignancy of Glioblastoma through PPRC1-ESM1 Axis Activation and Extracellular Matrix Remodeling**

Hsing-Fang Tsai^1*^, Yu-Chan Chang^2*^, Chien-Hsiu Li^1^, Ming-Hsien Chan^1^, Chi-Long Chen^3^, Wen-Chiuan Tsai^4#^ and Michael Hsiao^1,5#^

1. Genomics Research Center, Academia Sinica, Taipei, Taiwan
2. Department of Biomedical Imaging and Radiological Sciences, National Yang Ming Chiao Tung University, Taipei, Taiwan.
3. Department of Pathology, Taipei Medical University Hospital, Taipei Medical University, Taipei, Taiwan
4. Department of Pathology, Tri-Service General Hospital, National Defense Medical Center, Taipei, Taiwan
5. Department of Biochemistry, College of Medicine, Kaohsiung Medical University, Kaohsiung, Taiwan

*the first two authors contribute equally to this work

#To whom correspondence should be addressed:

Dr. Wen-Chiuan Tsai, Department of Pathology, Tri-Service General Hospital, National Defense Medical Center, Taipei, Taiwan, Tel: +886-2-8792-3100 ext. 13644, E-mail: [ab95057@hotmail.com](mailto:ab95057@hotmail.com). ORCID: 0000-0003-1085-9014

Dr. Michael Hsiao, Genomics Research Center, Academia Sinica, 128 Academia Rd., Sec. 2, Nankang-Dist., Taipei, Taiwan. Tel: +886-2-2787-1243, Fax: +886-2-2789-9931, E-mail: [mhsiao@gate.sinica.edu.tw](mailto:mhsiao@gate.sinica.edu.tw). ORCID:0000-0001-8529-9213

**Supplementary Figure and Figure Legends**

Supplementary Figure 1. The COL5A1 expression level is associated with poor survival in GBM patients. Kaplan–Meier plot of the overall survival rate of the Petel cohort based on high or low COL5A1 RNA expression levels (HR=3.97, *p*=2e-20).


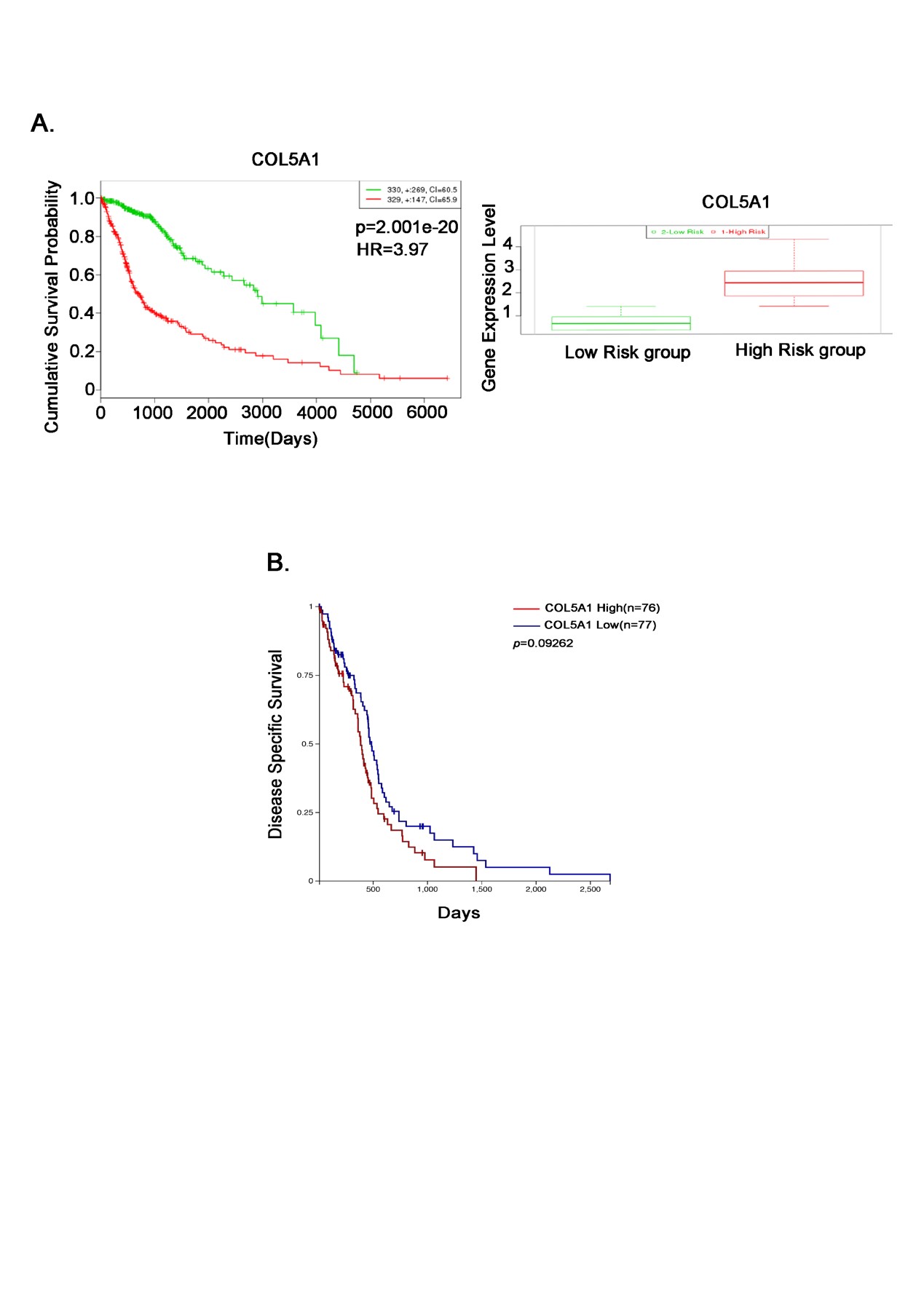


Supplementary Figure 2. Box plots show COL5A1 mRNA levels after the classification of several clinicopathological factors. The A.U. represents absorbance units.


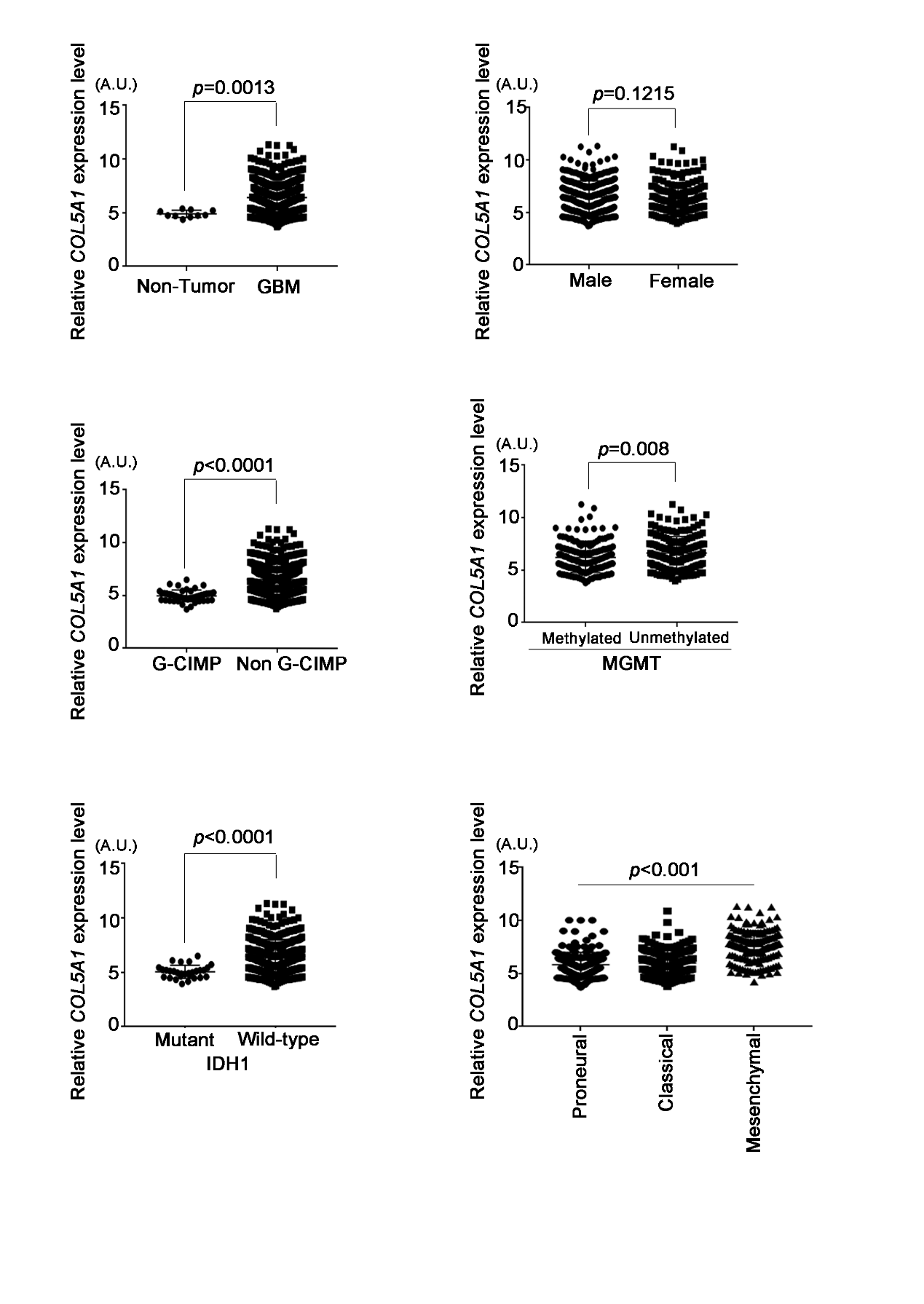


Supplementary Figure 3. Endogenous COL5A1 mRNA levels in GBM cancer cell lines from the Cancer Cell Line Encyclopedia (CCLE) database.


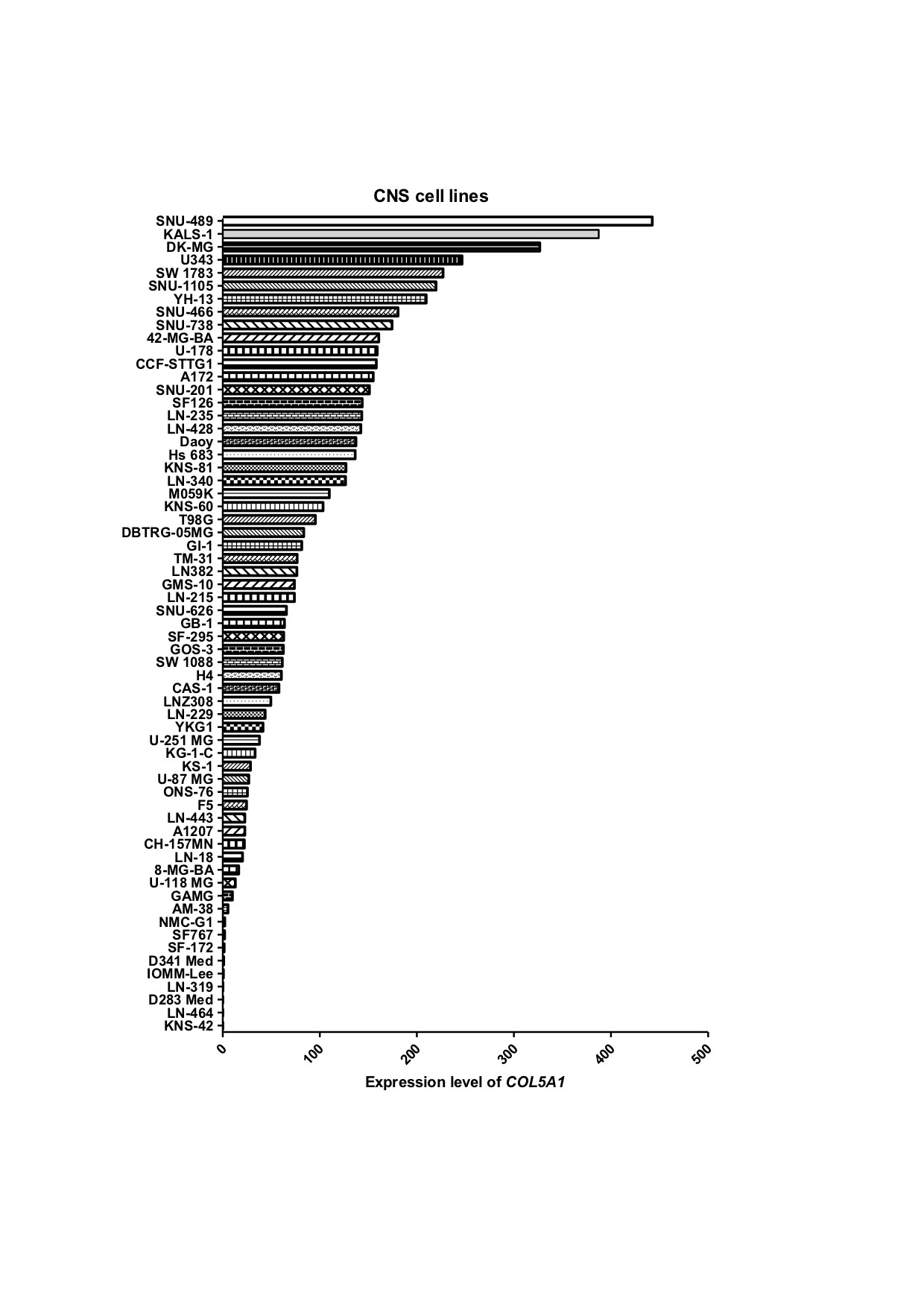


Supplementary Figure 4. The downstream targets of PPRC1 were analyzed by qRT–PCR in the A-172 COL5A1-knockdown model. The significance of the difference was analyzed using the nonparametric Mann–Whitney *U* test.


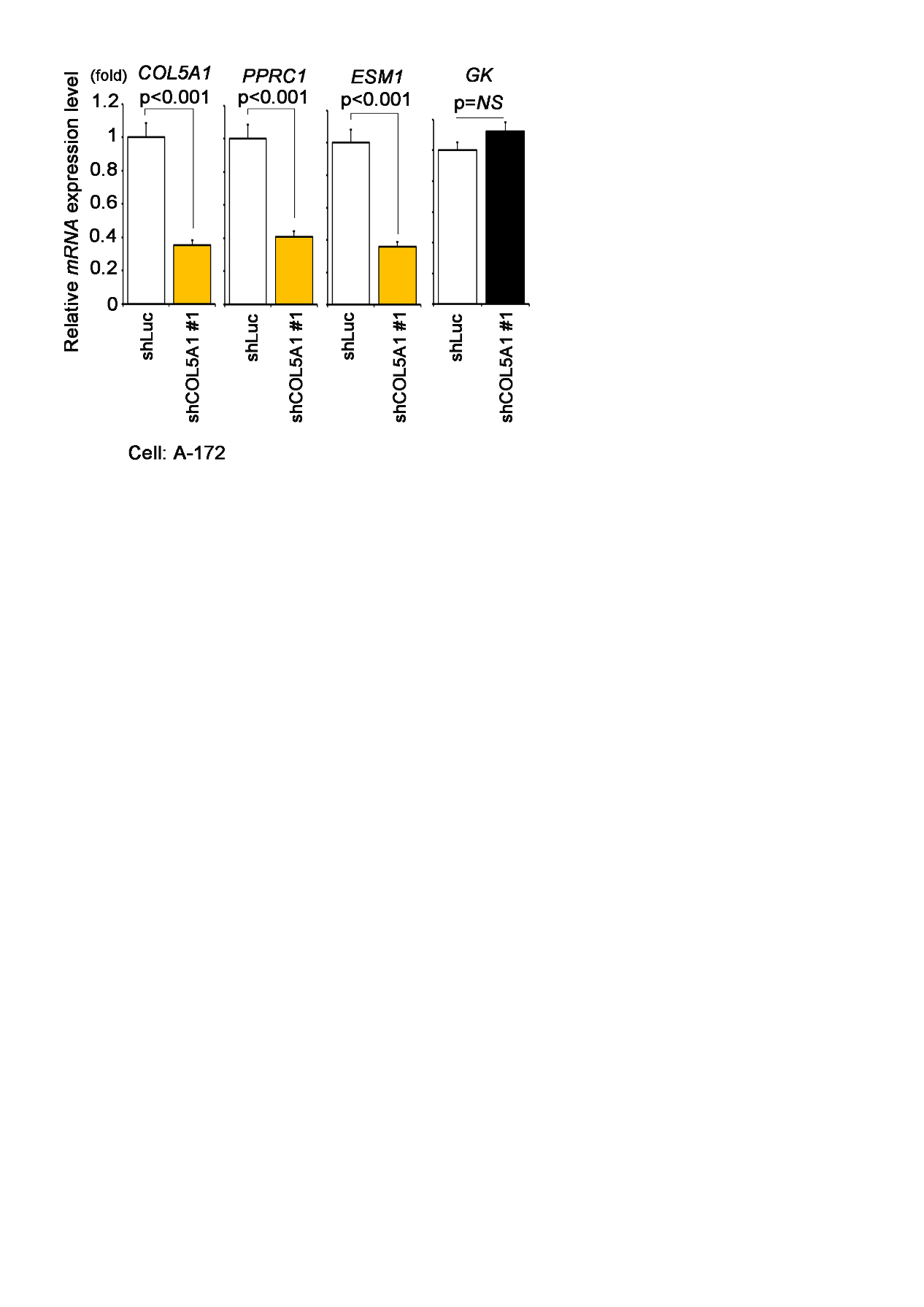


Supplementary Figure 5. The correlation between COL5A1 and PPRC1 downstream factors (CLDN1 and CCL20) in TCGA_GBM patients.


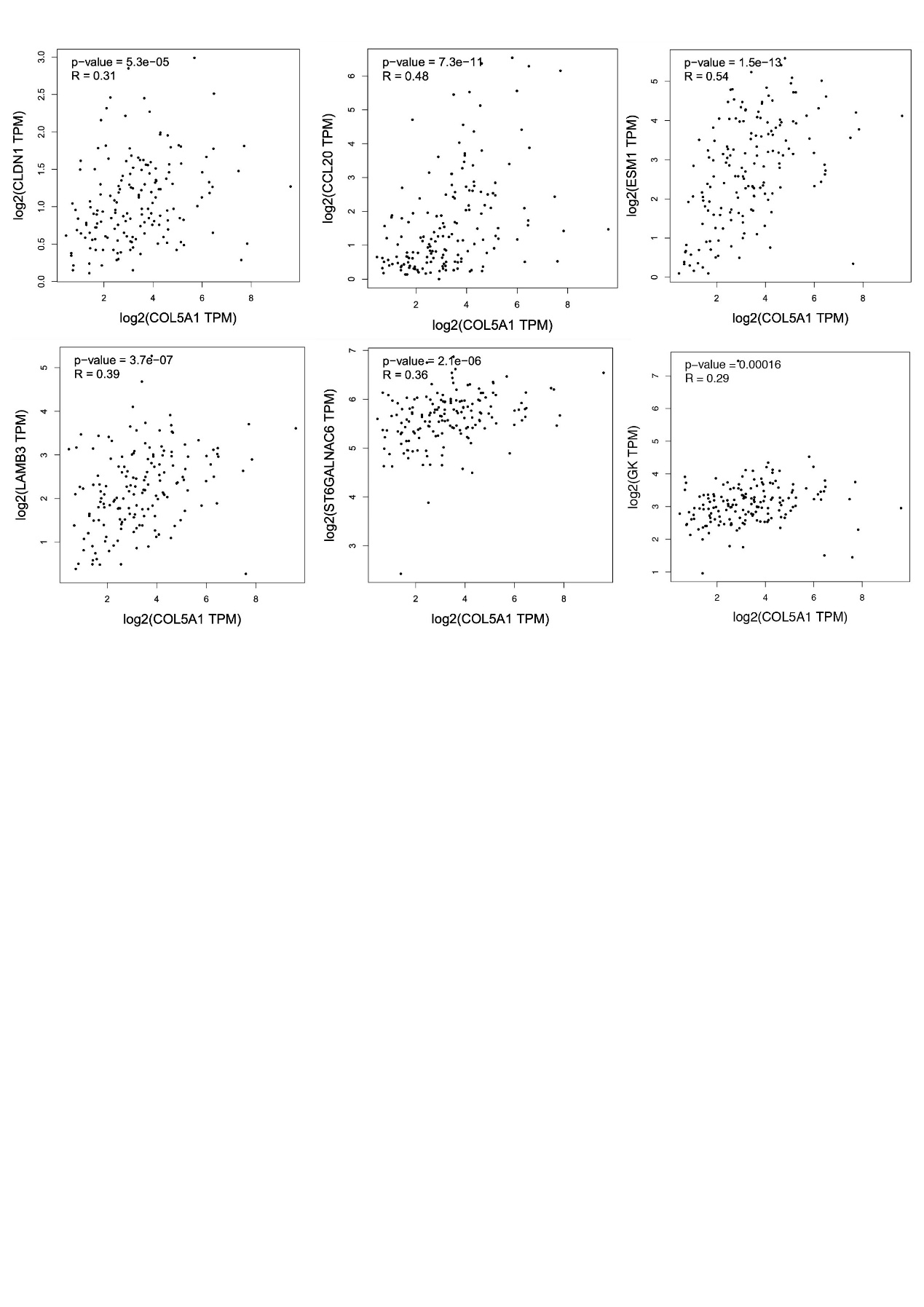

Supplement: Supplementary file 1 — Supplementary information [file 41420_2021_661_MOESM1_ESM.docx]
